# The three-dimensional impulse-response model: Modeling the training process in accordance with energy system-specific adaptation

**DOI:** 10.1371/journal.pone.0341721

**Published:** 2026-02-06

**Authors:** Hilkka Kontro, Armando Mastracci, Stephen S. Cheung, Martin J. MacInnis

**Affiliations:** 1 Faculty of Kinesiology, University of Calgary, Alberta, Canada; 2 Baron Biosystems Ltd., Toronto, Ontario, Canada; 3 Department of Kinesiology, Brock University, Ontario, Canada; University of Ljubljana, SLOVENIA

## Abstract

Athletic training is characterized by physiological systems responding to repeated exercise-induced stress, resulting in gradual alterations in the functional properties of these systems. The adaptive response leading to improved performance follows a remarkably predictable pattern that may be described by a systems model provided that training load can be accurately quantified and that the constants defining the training-performance relationship are known. While various impulse-response models have been proposed, they are inherently limited in reducing training stress (the impulse) into a single metric, assuming that the adaptive responses are independent of the type of training performed. This is despite ample evidence of markedly diverse acute and chronic responses to exercise of different intensities and durations. Herein, we propose an alternative, three-dimensional impulse-response model that uses three training load metrics as inputs and three performance metrics as outputs. These metrics, represented by a three-parameter critical power model, reflect the stress imposed on each of the three energy systems: the alactic (phosphocreatine/immediate) system; the lactic (glycolytic) system; and the aerobic (oxidative) system. The purpose of this article is to outline the scientific rationale and the practical implementation of the three-dimensional impulse-response model.

## Introduction

Of all means to improve athletic performance in humans, the most effective method is physical training. While this is a self-evident and universally accepted fact, substantial uncertainty remains around the type, volume, and intensity of training that athletes should complete to optimize performance for a given event [1–3] and the periodization approach athletes should follow to peak for competition [4,5]. Nevertheless, the main purpose of physical training is to induce physiological changes that result in more speed, strength, or fatigue-resistance, and depending on the specific goal of training, different modes and volumes of training are prescribed.

### Principles of adaptation

The improvements in performance following training are explained by the adaptive stimulus induced by exercise, whereby physiological systems respond to acute challenges in maintaining homeostasis [6,7]. When the stress experienced is high enough, it elicits an adaptive response in the form of altered gene expression and subsequent protein synthesis. On the other hand, this synthesis is preceded and counterbalanced by molecular and structural breakdown during exercise, and adaptation ultimately reflects the dynamic balance between these opposing processes [8]. The magnitude of the exercise-induced stress is—borrowing from physics—the “impulse” that is suggested to determine the magnitude of the adaptive response. The effect of these adaptive responses accumulates over time to produce functionally and structurally enhanced biological processes that, for example, facilitate a higher maximal or sustained energy turnover during muscular contractions [9]. Disruptions in homeostasis are greater the closer to a performance ceiling an athlete is exercising [10], meaning that higher intensities and longer durations generally lead to greater adaptive responses [11–13]. Depending on the task, a performance ceiling may exist due to a rate limitation or a capacity limitation in energy provision [14]. The importance and consequences of this distinction in training modeling will be discussed in later sections.

### Principles of modeling performance

Exercise physiology often employs a reductionist approach to explain how performance can be pinpointed to the individual components of the human body, such as muscle oxidative capacity, cardiac output, or neuromuscular activation. However, a holistic approach may also be adopted, in which the body is seen as an emergent system and performance arises from the integration of its components [15]. Contemporary physiology recognizes the integration of multiple interacting subsystems, including energetic, cardiovascular, neuromuscular, and endocrine-signaling processes, each of which contribute to the adaptive response. In line with this approach, mathematical models have been proposed to explain the changes in performance following training and cessation of training. In a systems model, performance (the output or response) can be predicted from the training completed (the input or impulse). Banister and Calvert were the first to introduce and validate a systems model to explain how performance changes in response to adjustments in training load [16,17]. After some initial models involving multiple components, they reduced their performance model to be a function of two variables: fitness and fatigue [17,18]. This model represented one complementary framework focused on quantifying the training–performance relationship rather than describing all mechanisms of adaptation. This impulse-response model, often referred to simply as the Banister model, survives to the present day and is, in various forms, the basis for current approaches to monitoring and predicting fitness, fatigue, and performance.

**Fig 1:**
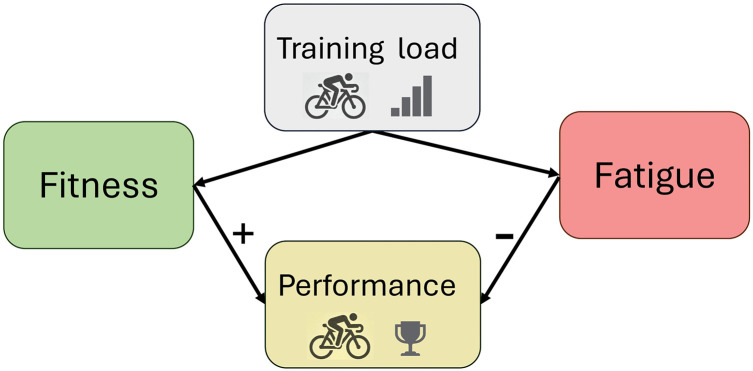
The Banister impulse-response model. Performance is a function of fitness and fatigue, which both increase in response to training, but by different magnitudes and following different timeframes. Adapted from Morton et al. (1990) [21].

The theory behind the Banister impulse-response model builds on the observation that while increases in training lead to improved fitness (the positive component), they also generate fatigue (the negative component). Performance at a given time point thus equals fitness minus fatigue (Fig 1).

## A novel power-based training load metric

In order to apply an impulse-response model to training, one of the fundamental premises is a valid metric for the input. Training load describes the dose of training and is the required input to model the dose-response relationship of training and adaptation. In this section, we introduce a novel way of analyzing power output-based training data.

### How can training load be quantified?

Training loads can be classified into external/absolute loads or internal/relative loads. External measures of load include objective measures such as duration, work, work rate, speed and distance. Conversely, internal loads reflect the training-induced stress experienced by the athlete [19]. While external training load is easily quantifiable, particularly in cycling with the widespread use of power meters, it is the magnitude of the internal load that is thought to initiate the adaptive responses to training [20]. Internal loads can be assessed using physiological and perceptual markers or by anchoring an external measure of work rate to individual exercise thresholds. In the following section, the existing methods will be briefly discussed.

We acknowledge that established terminology related to training load borrows terms from physics and applies different (albeit analogous) meanings to each term. Rather than create new terms, the present manuscript and model uses the terms load, stress, impulse, and strain outside of their correct use.

### Popular models for heart rate-derived training load

To quantify the dose of training based on heart rate (HR), a training load metric called training impulse (TRIMP) was introduced by Banister and colleagues [21]. It factors in both duration and HR relative to the individual’s HR reserve (maximal HR – resting HR). Briefly, Banister’s TRIMP is calculated from duration of exercise and the (expected) relationship between HR and blood lactate during an incremental test, multiplied with the HR reserve and a weighting factor [21]. The weighting factor is applied to avoid giving too much emphasis to low-intensity exercise of long duration relative to higher intensities. Alternative HR-based TRIMPs are Edward’s TRIMP, which is calculated as a sum of time spent in five HR zones (determined by %HRmax) multiplied by zone-specific weighting factors [22], and Lucia’s TRIMP, in which weighting factors are anchored to ventilatory thresholds instead of arbitrary HR zones [23], although the weighting factors remain somewhat arbitrary. These HR-based TRIMP training load metrics have been a central tool in both research and coaching [19].

Despite some investigations reporting satisfactory performance prediction of the impulse-response model when using the TRIMP concept, serious limitations of TRIMP remain. One of the underlying problems with TRIMP lies within the inherent limitations of using HR as an all-encompassing marker of intensity. The kinetics of the HR response (such as inertia and drift) as well as factors independent of power output (such as environmental conditions and level of arousal) impair its ability to accurately reflect muscular energy requirements, especially in very short, very long, and intermittent (i.e., on/off) efforts. As an example, Vermeire and colleagues analyzed the training of 11 recreational cyclists over 12 weeks and failed to observe a consistent relationship between different TRIMPs and changes in performance [24]. The lack of a consistent dose-response relationship between TRIMP and progression in physical fitness was attributed to limitations with the calculation and the general concept of the training load model used. In a later review article, the same research group discussed limitations of this impulse-response model and concluded that “since the training adaptations performing such different training sessions with a similar [training load] are totally different, the relationship with performance improvement will always be distorted” [25]. HR-derived training load metrics are no longer preferred for quantifying training loads and instead, the use of power meter data to generate a training load metric has become the standard in cycling.

### Popular models for power-derived training load

With the increasing affordability and validity of portable power meters over the last decade or two, power-based training load metrics have taken over as the most popular way to estimate training load in the field for cycling. Training Stress Score (TSS^®^; registered trademark by TrainingPeaks) is a training load metric derived from power output recordings [26]. This metric is anchored to the individual functional threshold power (FTP; an estimate of critical power or maximal lactate steady state) and, by definition, 1 hour at this threshold gives 100 TSS. Its calculation uses a concept called normalized power (NP^®^, also registered trademark by TrainingPeaks), which transforms raw power output values to NP by specific weighting of rolling 30-second average power output. This method aims to account for delayed physiological responses to changes in power output, and for their curvilinear rather than linear nature. TSS for a given training session is calculated as:


$$TSS=\frac{t\cdot NP\cdot IF}{FTP}\cdot \frac{\text{1}\text{0}\text{0}}{\text{3}\text{6}\text{0}\text{0}s}$$ (1)

where IF is the intensity factor and simply the fraction of NP relative to FTP (if NP = FTP, IF becomes 1.0, and 3600 is the number of seconds in an hour. This method has also been transformed to calculate TSS from HR data (for any activity) and from velocity data (for running and swimming) [27]. TSS has been demonstrated to correlate with performance increases equally or better when compared with various HR-derived TRIMPs [24,28,29]. In work by Sanders et al. [28] external load via TSS correlated strongly (r = 0.75–0.79) with changes in sub-maximal fitness (power at 2 and 4 mmol L ⁻ ¹) and moderately with an 8-min time trial. Wallace et al. [29] reported that TSS correlated (r = 0.70) with 1,500-m performance in runners, slightly better than TRIMP and session-RPE (r = 0.65 and 0.60, respectively). However, Vermeire et al. [24] found inconsistent associations between TSS, different TRIMP models, and performance in a 3-km cycle ergometer time trial.

While TSS might overcome some of the limitations of HR-based methods to quantify training load, it remains largely unvalidated against physiological measurements and has significant limitations that ultimately reduce its ability to predict adaptation. The first limitation, which is common to all other popular training load metrics, is the one-dimensional nature: adaptation is assumed to be independent of intensity. For example, this model assumes that performing all training at an IF of 0.5 would result in the exact same adaptations as performing all training at an IF of 1.1 (but spending ~80% less time doing so), which is not supported by practice or scientific literature.

Another limitation of TSS lies in the independence of duration in the calculation of NP. TSS only considers the distance from FTP when estimating metabolic stress, not the duration for which it is sustained. This is a problem particularly for efforts above FTP (non-steady state exercise). For instance, if we consider a maximal constant-load cycling bout of 20 minutes, the TSS calculated will be the same for minute 2 as it is for minute 19. However, metabolic stress will be close to maximal achievable levels by the end of the task, hence the adaptive stimulus of minute 19 is likely greater than that of minute 2. Indeed, high-intensity interval training relies on this concept; otherwise, there would be no benefit to doing repetitions that generate greater metabolic stress and strain. To overcome this limitation, a power-based training load metric must be able to estimate physiological strain with the help of the entire power-duration curve to assess how close to their maximum rates and capacities each energy system is operating at a given moment. The proposed training load metric in this paper (named strain score, SS), despite also being power-based, considers time spent above the maximal metabolic steady state to estimate the level of strain. The mathematical basis of calculating SS will be introduced in the next section.

## Three dimensions of training load

Muscles utilize three complementary energy systems to power muscle contractions. During even intense muscle contractions, the concentration of ATP stays relatively stable due to the instant replenishment of the ATP that is hydrolyzed mainly by contractile machinery and ion pumping. Resynthesis of ATP is achieved by 1) the alactic anaerobic (PCr/immediate) system; 2) the lactic anaerobic (glycolytic) system; and 3) the aerobic (oxidative) system, and the systems activate in this order, albeit in close succession, at the onset of exercise. While these systems remain active simultaneously with continued contractions, their relative contribution to energy production depends heavily on the duration and intensity of the exercise bout. For a comprehensive review on exercise energy metabolism, see Hargreaves & Spriet (2020) [30].

### Rationale for energy-system specific adaptation

Vast amounts of literature exist to demonstrate that training at different ends of the power-duration spectrum elicits different performance adaptations [31,32]. For example, 8 weeks of continuous running training at moderate intensities elicited improvements in aerobic capacity and running distance in a 30-min test, while short sprint training showed no improvements in these variables but did improve the 50 and 100 m sprint times [33]. Furthermore, a combination of both training types improved both the aerobic and the sprint outcomes [33]. Continuous or intermittent training relying heavily on the oxidative system is well-established to promote improvements in maximal aerobic capacity [34] and critical power (CP) [35].

In addition, performing all-out sprint interval training, such as 30-second sprints on a cycling ergometer, has been shown to improve both aerobic and anaerobic performance and increase the capacity of all systems [36,37]. In this form of exercise, despite the relatively short duration of the work bout, the oxidative system is fully activated at the end of the first sprint and contributes an increasingly greater share of the ATP in subsequent sprints, so that the muscle receives signals to augment all three energy systems [38,39]. Conversely, sprint training involving only very short sprint bouts (5–10 s) with long recovery has been shown to decrease [40] or lead to no change [41] in markers of oxidative capacity, in line with its low dependence on the aerobic system to provide ATP for the task.

As there is evidence of the three energy systems responding independently with specific adaptations to each, it could be appropriate to model the “fitness” of each system independently rather than using a global amalgamation of them, which inevitably results in compromising their true attributes for simplicity. By quantifying training loads across these three systems, our proposed approach separates each system, resulting in three parallel impulse-response models, each corresponding to an energy system.

### From energy systems to the power-duration relationship

What are the limiters for instant power output and sustained power output and why should they be considered in performance modeling? Individual endurance exercise capacity at a given intensity can be predicted from a series of maximal efforts in the severe exercise domain [42]. The power-duration relationship for cycling is generally accepted to be hyperbolic, and its asymptote is critical power (CP) [43]. The CP model was originally proposed for single muscle group exercise [44] but was later adapted to describe power-duration behaviour in whole body exercise [45,46].

CP has been suggested as the gold standard delineating the heavy intensity domain from the severe intensity domain [47]. As such, CP is the ceiling for the rate of sustainable oxidative energy provision, as it separates exercise intensities for which a metabolic steady state (i.e., stable oxygen uptake) is achievable from intensities for which it is not [10,48]. According to the standard interpretation of the CP model, exercising above CP results in the gradual depletion of W′—the finite work capacity above CP—until task failure occurs, whereas exercising below CP does not draw on W′, implying (mathematically) that exercise below CP can be performed indefinitely [42]. Despite its popularity, this 2-parameter model incorrectly assumes that when duration approaches zero, power output approaches infinity. A 3-parameter (3-CP) power duration-model includes maximal sprint power (Pmax) as a third parameter, addressing the unrealistic assumption of unlimited W′ utilization rates [49].

The equation defining the popular 2-parameter power-duration relationship is:


$$t_{lim}=\frac{W^{\prime }}{P-CP}$$ (2)

This CP model assumes an unlimited rate of W′ expenditure. A 3-parameter (3-CP) power duration-model that includes Pmax as a third parameter has been shown to result in better fit for short exercise durations [49,50]. It is defined as:


$$t_{lim}=\frac{W^{\prime }}{P-CP}-\frac{W^{\prime }}{Pmax-CP}$$ (3)

where Pmax represents the highest instant power that can be produced for a very short duration and is reached at an intensity at which the alactic/PCr system is providing most the ATP.

A consequence of the 3-parameter model is that the theoretical Pmax is only achievable in a fatigue-free state [14,51]. The highest possible power output, maximum power available (MPA), is a function of the remaining W′ and decreases with W′ expenditure (W′exp). Therefore, MPA changes according to the equation:


$$MPA=Pmax-(Pmax-CP)\cdot \frac{W^{\prime }exp}{W^{\prime }}$$ (4)

where W′exp = W′ - W′bal and W′bal is the amount of W′ remaining at a given moment. For constant power outputs P > CP, task failure occurs when P = MPA, not when W′ is fully depleted.

The three parameters (CP, W′, and Pmax) and the power-duration curve they define can be interpreted to contain information of the three energy systems: their maximal rates and their capacities (for the anaerobic systems, since the aerobic system is not considered capacity-limited) [14]. Consequently, a 3-parameter CP model may be used to estimate the contribution of each energy system to the power output at a given moment in time. This is, of course, a simplified assumption: the model is not meant to accurately quantify ATP fluxes through these systems, but to provide a crude estimate of energy provided by each, perhaps adequate for training load-related analyses.

**Fig 2:**
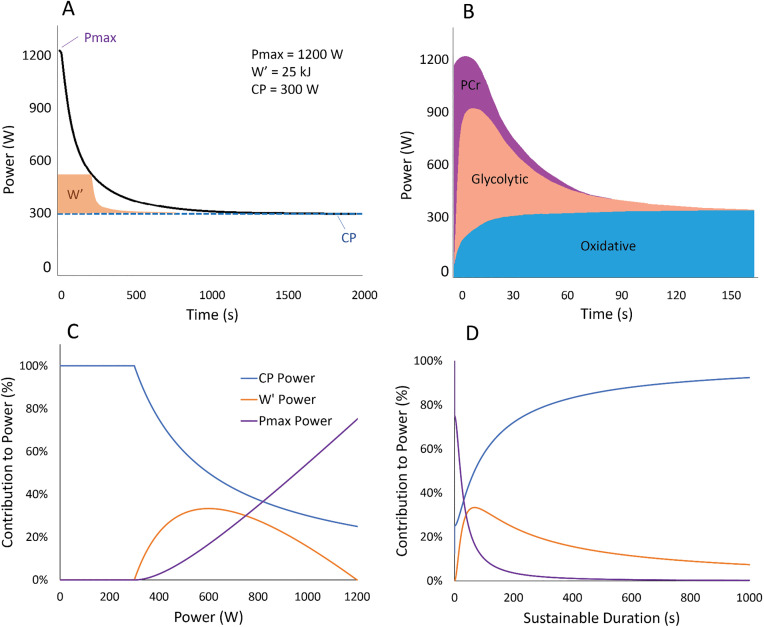
Linking the three energy systems with the three parameters of the power duration relationship. **A)** The power output attainable for a given duration can be predicted from a 3-parameter critical power (CP) model, in which maximal power output is limited by Pmax, highest aerobically sustainable power output is limited by CP, and the finite work capacity above CP, curvature constant (W′), is primary limited by buffering capacity and muscle energy stores available for substrate-level phosphorylation. **B)** A schematic representation depicting the contribution of each energy system to power production in all-out exercise of 2.5 minutes. The relative contribution of each system is related to the parameters Pmax (the PCr system), W′ (the glycolytic system), and CP (the oxidative system). Constructed based on data from Baker et al., (2010), Morton (2006), and Vanhatalo et al., (2007) [14,52,53]. **C)** The contribution of the parameters of the 3-parameter CP model to a given power output plotted against power. **D)** The contribution of the parameters of the 3-parameter CP model to a given power output plotted against the maximal sustainable constant-load duration **(D)**.

The connection between the three parameters of the power-duration relationship and the energy systems is illustrated in Fig 2 (A, B).

### Quantification of energy system contributions

Continuous power data may be converted to energy system-specific work based on power output and how close the individual is to their MPA at a given moment in time. Furthermore, these values may be used as input values for training load imposed on each energy system. The theoretical basis of this conversion is described below.

At a given moment of time, the power output (*P*) is a sum of energy provision by each energy system. For example, the oxidative system (represented by CP) is unable to provide enough ATP to power 600 W; therefore, some of the energy must come from the alactic system (Pmax) and the lactic system (W′). How much energy comes from each system for each athlete? When an athlete’s CP and Pmax are known, we may mathematically derive the contribution of each system to a given *P*. Using the MPA formula (Equation 4), we compute a power duration relationship for a constant power performed to failure in the special case where *P* = *MPA*:


$$P=Pmax-(Pmax-CP)\cdot \frac{(P-CP)t}{W^{\prime }}$$ (5)

This is the constant power sustainable for time *t*. Solving for *P*:


$$P=CP+\frac{(Pmax-CP)W^{\prime }}{W^{\prime }+(Pmax-CP)t}$$ (6)

We now have the power-duration formula for the 3-parameter CP model. We can rearrange this formula to compute the contributions of CP, Pmax and W′ to power P, as a function of CP, W′, Pmax and P by separating into 3 separate terms:


$$P=CP+\frac{{\left(P-CP\right)}^{\text{2}}}{Pmax-CP}+\frac{{\left(P-CP\right)}^{\text{2}}t}{W^{\prime }}$$ (7)

From this basis, the contributions of CP, Pmax and W′ may be derived, assuming *CP* represents the maximum rate of aerobic power. Therefore:

Contribution of *CP*:


$${If\;P<CP,\;P}_{CP}=P;If\;P>CP,P_{CP}=CP$$ (8)

Contribution of *Pmax:*


$$P_{Pmax}=\frac{({P-CP)}^{\text{2}}}{Pmax-CP},\;\text{where}\;P>CP$$ (9)

Contribution of *W′:*


$$P_{W^{\prime }}=\frac{{\left(P-CP\right)}^{\text{2}}}{W^{\prime }}=P-CP-P_{Pmax},\;\text{where}\;P>CP$$ (10)

Equation (9) can be understood through the following reasoning. When exercising at CP, Pmax (alactic system) has no contribution to power, since the aerobic system can fully account for the ATP turnover. In contrast, when exercising at Pmax, Pmax has 100% contribution to the power (above CP), as instant maximal power is not limited by W′. Therefore, between CP and Pmax, the contribution of Pmax must get a value between 0 and 1. Equation (10) represents what must be left for W′ (lactic system) to contribute after subtracting the effects of CP’s and Pmax’s contributions. Finally, Equation (8) can be understood as the aerobic system contributing fully to all power up to CP, but no more to any increases in power above it.

To demonstrate the calculation: if the athlete with a CP of 300 W and Pmax of 1200 W is sprinting at Pmax, then all of the power generated that is above CP is attributed to Pmax. Any power output between CP and Pmax will have some contribution from all three parameters. If they are exercising at 1000 W, according to equations (8) to (10), 544 W is attributed to Pmax, 156 W to W′, and 300 W to CP. If exercising at 400 W, then the shares of power production from the three systems become 11 W, 89 W, and 300 W, respectively. If cycling at CP or below, 100% of power output is attributed to CP. The relative contributions of the parameters CP, W′, and Pmax, calculated using equations (8), (9), and (10), are illustrated in Fig 2 as plotted against power (2C) and against sustainable duration (2D).

How can these power allocations be translated into a training load input reflecting the share of each parameter accurately? As argued above, training-induced strain is a function of not only intensity, but also duration. Here, the concept of MPA becomes important, because it allows us to assess how close to their limit the individual is exercising at a given moment of time. When cycling at power output P (>CP) for long enough to deplete most of W′, MPA will approach P, making exercise increasingly difficult and elevating the strain induced by each second of additional exercise. We may estimate the strain coefficient (k_strain_, unitless) by:


$$k_{strain}=\frac{Pmax-MPA+CP}{Pmax-P+CP}$$ (11)

Strain rate (SR) is calculated by multiplying k_strain_ by power output (*P*):


$$SR=k_{strain}\cdot P$$ (12)

The SR (units: W) is calculated on a second-by-second basis for continuously recorded power data.

Finally, strain score (SS) quantifies how much total strain an athlete endures during an activity. To allow for comparison to TSS with values of similar magnitude, second-by-second SR is normalized to CP so that one-hour (3600 s) at CP equates to 100 SS.

*SS* is calculated as:


$$SS=\sum SR\cdot \left(\frac{Pmax}{{CP}^{\text{2}}}\cdot \frac{\text{1}\text{0}\text{0}}{\text{3}\text{6}\text{0}\text{0}s}\right)$$ (13)

The factor *Pmax/ CP*^*2*^ is used to achieve 100 *SS*/h when exercising at *CP*, as can be demonstrated by substituting the equations for *SR* (12) and *k*_*strain*_ (11) into Equation (13).

This SS can be subdivided into SS_Pmax_, SS_W′_, and SS_CP_ by using the system-specific strain rates SR_CP_, SR_W′_, and SR_Pmax_, calculated from the respective power contributions P_Pmax_, P_W′_, and P_CP_ (Equations 8–10).

**Fig 3:**
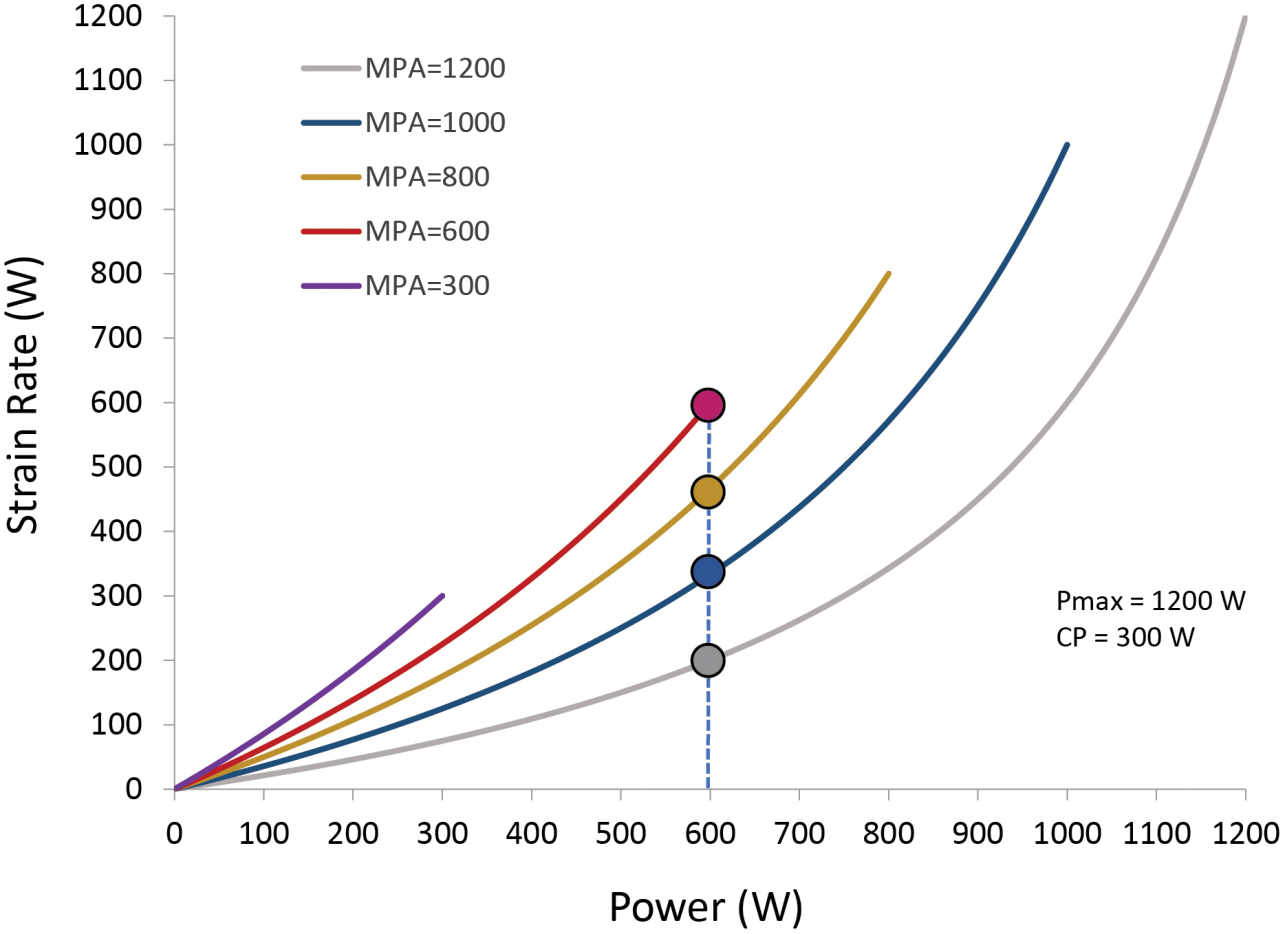
Maximum power available (MPA) influences the strain experienced at a given power output. MPA is a function of the amount of W′ expended. To generate 600 W when fresh (MPA = Pmax = 1200 **W)**, is less strenuous (grey dot) as compared to a situation where MPA is reduced to 1000, 800, or 600 W (blue, yellow, and red dot, respectively). When MPA = CP = 300 W, W′ is fully depleted and generating 600 W is not possible.

For example, if exercising fresh (i.e., W′exp = 0; see Equation (4)), and power P equals CP, MPA = Pmax and k_strain_ gets a value of 0.27. However, when available W′ is fully exhausted (W′exp = W′), MPA = CP and k_strain_ becomes 1.0. For this example athlete, the model implies that exercising at CP is approximately four times more strenuous when W′ has been depleted compared with when they were fresh. The impact of falling MPA on strain is illustrated in Fig 3.

### Advantages of the novel strain score

What practical advantages does the novel training load metric SS provide over existing metrics such as total work or TSS? First, since it considers duration of exercise above CP in addition to the distance from CP, it may more accurately estimate the metabolic stress, and consequently strain, than other power-based metrics.

**Fig 4:**
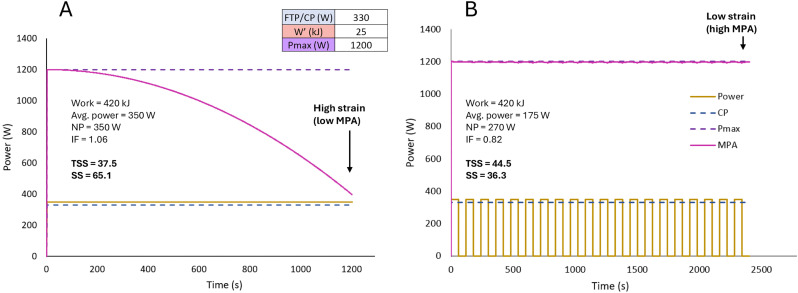
Limitations of using training stress score (TSS) to estimate the load of a session. **A)** A 20-min continuous effort at 350 W and associated training load metrics. **B)** A 20x1-min interval session at 350 W with 1-min passive rest. The example athlete’s parameters are listed in panel **A.** In both A and B, work completed is the same. TSS for scenario B is higher than for A despite greater expected metabolic perturbations in **A.** Strain score (SS) is higher for scenario A than for B, better reflecting the expected physiological strain of these efforts. At task failure, MPA (maximum power available) equals task power output. FTP = functional threshold power; CP = critical power; W′ = work prime; Pmax = maximal power output; NP = normalized power; IF = intensity factor. FTP and CP are assumed to be equal.

A comparison against TSS and work completed is illustrated in Fig 4.

Second, the SS metric introduced has three dimensions, allowing for a more specific quantification of training loads imposed on each energy system. Of note, this dimensionality should not be taken literally: the 3-dimensional impulse–response model operates in an abstract performance space rather than a physical one. In this space, each axis (x, y, z) represents one power-duration parameter (i.e., CP, W′, Pmax). A given performance state could then be visualized as a point within this coordinate system, whose position evolves over time in response to training stimuli.

**Fig 5:**
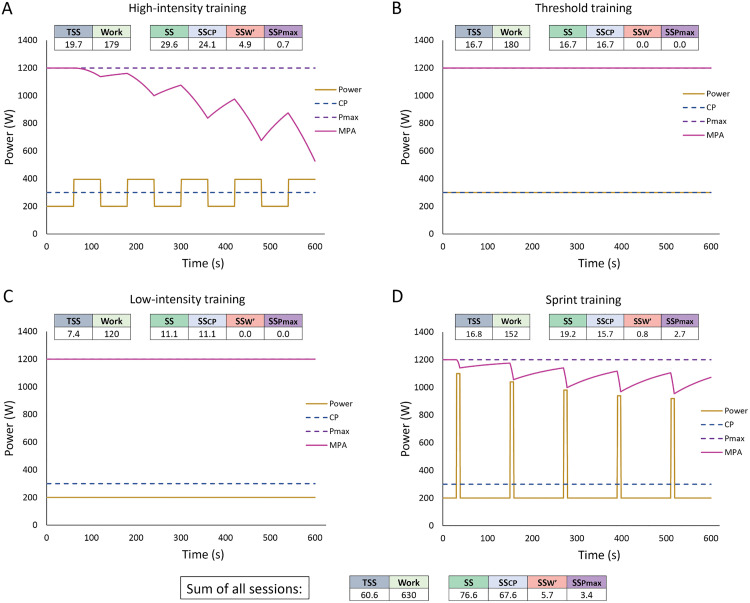
A comparison of total work, training stress score (TSS), and strain score (SS) in four different cycling sessions of 10 minutes. Despite the different types of training sessions, the TSS values resulting for A, B, and D are similar. SSCP, SSW′, and SSPmax represent the strain on the aerobic, glycolytic, and PCr energy systems, respectively, which reflects the training load of critical power (CP), work prime (W′), and maximal power (Pmax), respectively. The breakdown of SS into three dimensions allows for a more detailed analysis of the type of training performed. MPA = maximum power available, CP = critical power; W′ = work prime; Pmax = maximal power output.

This 3D feature helps overcome the well-recognized limitations of relying on one universal training load metric to encompass all training sessions of different types, and the resulting inaccurate predictions when using an impulse-response model. Nuances of training are not captured if using metrics such as TRIMP, TSS, or total work since they reduce vastly different training responses to a single value. Fig 5 illustrates the effect of quantifying training load using SS_CP_, SS_W′_, and SS_Pmax_ universal, one-dimensional metrics. Note that in Fig 4 and 5, the MPA shown follows the modified 3-parameter model where the W′exp/ W′ factor (see Eq 4) is raised to the 2^nd^ power [54] and the recovery of W′ balance follows the differential model by Skiba et al. (2015) [55] as the combination of these models have shown to effectively predict intermittent exercise in practice (unpublished data).

## The three-dimensional impulse-response model

The three training load metrics described in the previous section can separately be used to model performance across intensities and durations. The rationale to do this relies on the well-established properties of the power-duration relationship of exercise [14,56] as well as the principle of specificity, which is explained by the tendency of adaptation to only occur in the systems stressed and not others.

First, a brief introduction to the standard, one-dimensional impulse-response model is given. Consider the equation for performance by Morton et al. (1990) [21], who used this model to predict running performance:


$$p(t)=k_{\text{1}}g(t)-k_{\text{2}}h(t)$$ (14)

Where *p* is performance response, *t* is time (in days), *k*_*1*_ and *k*_*2*_ are weighting factors for fitness and fatigue, respectively, *g(t)* is fitness, and *h(t)* is fatigue. The values of *g(t)* and *h(t)* depend on the stimulus (i.e., training load), denoted below as *w(t)*, and the time interval, *i*, at which the stimulus is repeated:


$$g(t)=g(t-i)e^{-i/\tau_{\text{1}}}+w(t)$$ (15)

$$h(t)=h(t-i)e^{-i/\tau_{\text{2}}}+w(t)$$ (16)

**Fig 6:**
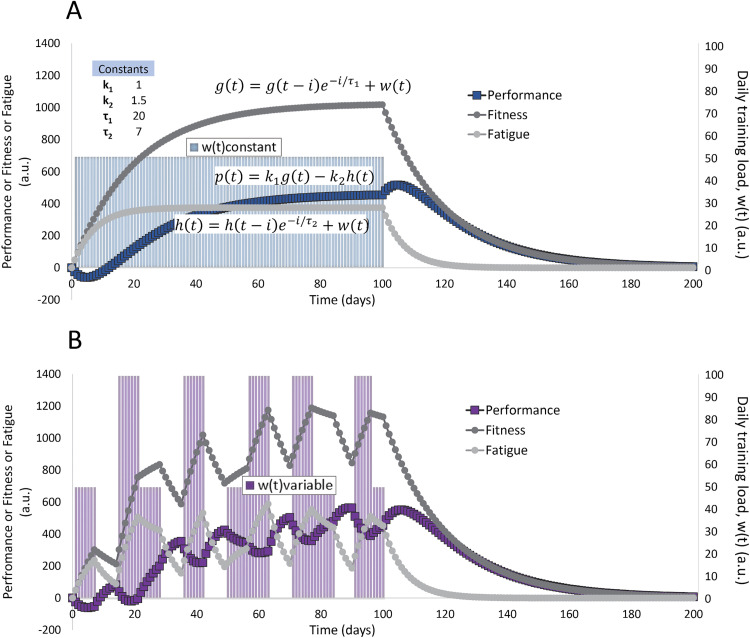
Example of predicted progression of fitness (g), fatigue (h), and performance (p). The simulation assumes 100 days of daily training at a constant (A) or variable (B) average training load of w(t) = 50 followed by 100 days of detraining at w(t) = 0, modelled using equations 14, 15, and16. Values for the weighting factors k1 and k2 are 1 and 1.5, respectively, and the time constants τ1 and τ2 are 20 days and 7 days, respectively. Units for g, h, and p on the y-axis are arbitrary. Despite an overall identical accumulated training load of 5000 units over the training period, the performance peak in the variable program (B) is greater than that of the constant program **(A)**.

The time constants *τ*_*1*_ and *τ*_*2*_ define the kinetics of fitness and fatigue progression, respectively. An optimal taper for peak performance involves manipulating the training load so that the loss in fitness is minimized while the loss in fatigue is maximized (the effect of varying training load on predicted performance is illustrated in Fig 6).

Popular training platforms use a slight variation of Banister’s original formula, where fitness is calculated as an exponentially weighted moving average of daily training load:


$$g(t)=g(t-\text{1})e^{-\text{1}/\tau_{\text{1}}}+w(t)(\text{1}-e^{-\text{1}/\tau_{\text{1}}})$$ (17)

Or, using popularized terminology:


$$CTL={CTL}_{yesterday}e^{-\text{1}/\tau_{\text{1}}}+{TSS}_{today}(\text{1}-e^{-\text{1}/\tau_{\text{1}}})$$ (18)

Where CTL stands for chronic training load and is equivalent to fitness.

With varying success, the impulse-response model has been used to relate training load to changes in performance in different sports, including swimming [17,57,58], triathlon [59], cycling [24,60,61], and running [21,29,62]. However, substantial uncertainty remains around the model’s ability to predict performance, which might be in part explained by the difficulty of quantifying training load as outlined in the prior sections.

### Implementation of the 3D-impulse-response model

As detailed in section III, the new training load metric SS makes use of continuous power meter data to convert second-by-second work to strain based on an athlete’s proximity to their MPA [54,63]. Second-by-second strain for each fitness parameter can be calculated by multiplying strain rate with the ratio corresponding to the contribution of each energy system (Table 1).

**Table 1:** Example of the strain score calculation for different levels of MPA.

	MPA = 1200	MPA = 1000	MPA = 800	MPA = 600	MPA = 400
Power output (W)	**k**_**strain**_				
100	0.21	0.36	0.50	0.64	0.79
200	0.23	0.38	0.54	0.69	0.85
300	0.25	0.42	0.58	0.75	0.92
400	0.27	0.45	0.64	0.82	1.00
800	0.43	0.71	1.00		
1200	1.00				
Power output (W)	**SR (W)**				
100	21	36	50	64	79
200	46	77	108	138	169
300	75	125	175	225	275
400	109	182	255	327	400
800	343	571	800		
1200	1200				
Power output (W)	**SS (∙ s** ^**-1**^ **)**				
100	0.008	0.013	0.019	0.024	0.029
200	0.017	0.028	0.040	0.051	0.063
300	0.028	0.046	0.065	0.083	0.102
400	0.040	0.067	0.094	0.121	0.148
800	0.127	0.212	0.296		
1200	0.444				
Power output (W)	**SS (∙ h** ^**-1**^ **)**				
100	29	48	67	86	105
200	62	103	144	185	226
300	100	167	233	300	367
400	145	242	339	436	533
800	457	762	1067		
1200	1600				

Note: Calculated for an athlete whose CP = 300 W and Pmax = 1200 W.

MPA = Maximum power available; k_strain_ = strain coefficient, SR = strain rate; SS = strain score.

**Fig 7:**
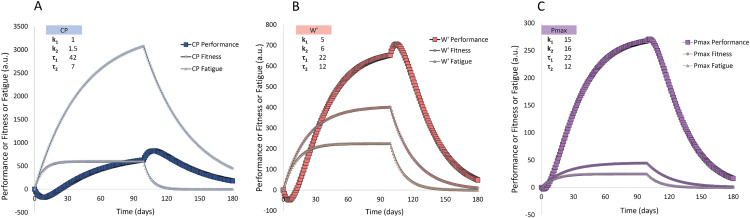
A hypothetical example of how a three-dimensional impulse model predicts changes in performance. Performance of the oxidative system **(A)**, the glycolytic system **(B)**, and the PCr system (C) changes based on changes in fitness and fatigue for each system. The performance outcome (a.u.) at a given timepoint can be translated into critical power (CP), work prime (W′), and maximal power output (Pmax) using a conversion factor to get units of W, kJ, and W, respectively. Parameter values for the model (weighting factors k1 and k2 and the time constants τ1 and τ2) can be experimentally determined for each system. In this simulation, w(t) is repeated daily and is 80, 18, and 2, for CP, W’, and Pmax, respectively.

When calculated in this manner, training load (w(t)) is divided into three components and fed into three parallel impulse-response models. This way, the three-dimensional model produces three separate performance management charts (PMCs) instead of one. Fig 7 shows a simulation of how fitness, fatigue, and performance of each system could evolve in response to a daily load of w(t) = 80, w(t) = 18, and w(t) = 2 (A.U.) for the oxidative (CP), glycolytic (W′), and PCr (Pmax) systems, respectively.

**Fig 8:**
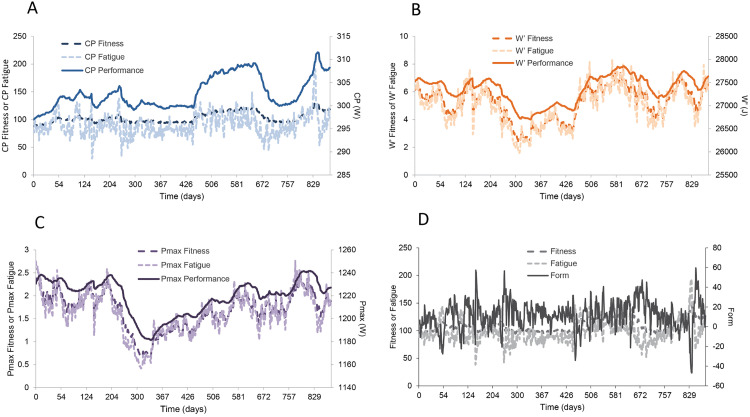
An example of an athlete’s training data collected over~2.5 years visualized in a performance management chart (PMC). **A)** One-dimensional PMC with overall fitness, fatigue, and form (performance readiness). Panels B, C, and D illustrate the three-dimensional model and the independent evolution of critical power, W′, and Pmax, respectively, over the same period.

Translation of the arbitrary units for fitness to directly informative units of power and work is preferable, as these fitness markers represent the parameters of the 3-parameter CP model. An example of real training data when visualized either in a traditional performance-management chart (PMC) or the three-dimensional model is shown in Fig 8. Notably, the three parameters CP, W′, and Pmax, do not always evolve in the same direction with increases or decreases in overall training load, but they respond in distinct ways depending on the type of training performed. An example of how training data can be analyzed is given in Supplementary material 1. An associated R code is provided for data processing.

### What are the model parameters for each energy system?

It is expected that the parameters (weighting factors, k, and time constants, τ) for the adaptation of each energy system are different. While no published data exist to support the energy-system specific model parameters, the assumption of their independence is suggested by data demonstrating that anaerobic performance can be enhanced in shorter time frames (different τ) and with much smaller doses (different k) than aerobic performance [36,64–66]. This is well-established in practice, which is why effective training plans have historically used a periodization strategy of more high-volume, low-intensity training early in the season and increasing amounts of high-intensity training closer to competition [67,68]. A recent meta-analysis suggests that while improvements in aerobic capacity can be achieved with sprint intervals, high-intensity intervals, or continuous exercise, the time-course of change is proportional to intensity (i.e., slower for lower intensities) [64]. In addition, the gains in VO_2max_ are greater with high-intensity intervals and continuous exercise training than with sprint interval training, which is consistent with their greater accumulated stress on the aerobic system [64]. This evidence suggests that emphasizing higher intensities (as opposed to moderate/ threshold intensities) closer to the competition date is beneficial for maximizing high-intensity performance rapidly, but the aerobic system may be trained over longer time frames.

The weighting factors (k) and time constants (τ) in the literature, both for fitness and fatigue, are based on a one-dimensional impulse-response model. Therefore, the reported parameters are reflecting some type of average of the performance of the responses of different energy systems. This might not be of great significance in sports where one energy system is clearly dominant and almost exclusively responsible for the observed changes in performance (e.g., the oxidative system in marathon running, or the alactic anaerobic system in weightlifting), and most training is also accumulated in the same intensity domain. However, countless sports (e.g., track cycling, track running, swimming, rowing, speed skating) involve maximal efforts spanning a range of less than one minute to several minutes and/or are highly stochastic in power demands. In addition, sports like team sports or road cycling combine long durations of low-intensity exercise with intermittent high-intensity efforts. Therefore, in most sports, successful athletes do not rely only on their CP, as W’ and Pmax substantially contribute to their competitive success. In these sports, modeling fitness and fatigue of the energy systems separately is expected to produce a more complete picture of athletic capabilities spanning a wide range of durations and intermittent exercise performance.

The model parameters are individualized to the athletes as training responsiveness shows large inter-individual variation [69,70]. This variation has been suggested to arise from variation in factors including genetics [71], diet [72], indicators of stress [73], initial fitness level [74], and generic training prescription leading to unequal internal load between individuals [75]. Ideally, the values for τ and k are established individually for each athlete and recalibrated periodically. In practice, training platforms use fixed parameters and the τ parameter is often estimated to be 42 days for fitness and 7 days for fatigue. Values for k cannot be compared across models using different training load inputs (which are in arbitrary units not easily transformed to one another), but time constants are unlikely to depend on the training load metric. Table 2 summarizes some time constants reported in the literature.

**Table 2:** Reported time constants for fitness (τ_1_) and fatigue (τ_2_) in endurance sports.

Study	τ_1_	τ_2_
Busso et al. (2003) (Period 1)	41 ± 15	9 ± 6
Busso et al. (2003) (Period 2)	35 ± 12	13 ± 3
Chalencon et al. (2015) (Banister model)	51 ± 14	10 ± 9
Chalencon et al. (2015) (Variable dose-response model)	48 ± 16	8 ± 4
Morton et al. (1990) (Subject 1)	50	11
Morton et al. (1990) (Subject 2)	40	11

## Limitations

While addressing some limitations inherent to the simple Banister model, the three-dimensional model described above is not free from limitations. First of all, several assumptions, some of which are acknowledged to be unrealistic, must be made to keep the model sufficiently simple to be useful. The underlying assumptions are as follows:

1) Each energy system adapts in proportion to its contribution to energy turnover, amplified by the magnitude of strain.
2) The adaptive process of an energy system can be uniformly described by simple parameters.
3) The fitness weighing factor (constant k_1_) does not change over time.
4) The contribution of each energy system can be derived from a three-parameter critical power model.
5) At the onset of exercise, energy provided by the aerobic system is immediately available at a maximum rate.
6) The energy supply by each energy system remains constant at a given power output.
7) There is no change in efficiency over the course of an exercise bout.
8) The power-duration parameters CP, W′, and Pmax do not change over the course of exercise.

Of the listed assumptions, number 1 is currently unknown, as the presented model has not been under strict scientific scrutiny. Assumptions 2 and 3 are common to all Banister models. Number 2 is an inherent quality of a systems model, which a reductionist approach could argue against. For example, both mitochondrial and cardiac adaptations may contribute to the fitness of the aerobic system, but their time course of change is very different despite the model using a single time constant [9,76]. Number 3 is unknown but possibly false, as there likely is some maximum to physiological qualities that cannot respond further by infinite increases in the stimulus – but whether this maximum is often approached in practice is uncertain. Number 4 has also not been experimentally confirmed despite a solid theoretical basis; however, it is acknowledged that assumptions 5–7 skew the calculation of energy system-specific contributions if not corrected (not implemented in this model). Numbers 5 and 6 are false based on the well-described kinetics of the V̇O_2_ response and the delayed contribution of the glycolytic system relative to the PCr system [77]. Despite this fact, the oxygen deficit created at the onset of exercise—and erroneously ascribed to the aerobic system in the model—will eventually be repaid by the aerobic system; therefore, this simplification may not reduce the accuracy considerably. Assumption number 8 is also inaccurate in the light of emerging evidence, and the parameter most affected might be W′, which is sensitive to glycogen availability [78].

Despite its limitations, the model presented is an attempt to synthesize current understanding of adaptive responses and the principle of specificity with a systems model. While we believe it to be an improvement to existing models, we also acknowledge the lack of experimental data to assess its performance. The model predicts future “fitness signatures” (i.e., the parameters CP, W′, and Pmax), which means predicting points of failure during maximal exercise. Each parameter in the model can be viewed as a functional index that predominantly reflects the capacity or rate limitations of different energetic domains. However, these variables should not be strictly interpreted as isolated ‘systems’; rather, they emerge from the collective behavior of metabolic, cardiovascular, and neuromuscular processes operating on overlapping time scales.

To experimentally validate the model, as done by previous researchers with other models, a performance prediction from a carefully quantified training block needs to be compared with performance outcomes. Given the popularity of power meters, the ability to analyze big datasets from real-life training may prove helpful in fulfilling this goal.

## Conclusions

The three-dimensional impulse-response model presented in this paper has some improvements compared to the simple Banister model. A one-dimensional model is widely used in endurance sports—particularly in cycling—despite its acknowledged shortcomings. By quantifying training load as three separate input metrics in the model, each reflecting the fitness of their respective energy system, the training principle of specificity is accounted for, theoretically resulting in more precise performance predictions. In addition, the quantification of training loads using the MPA concept, derived from a three-parameter power-duration model, appropriately considers exercise duration and not intensity alone as a variable affecting the level of metabolic stress during non-steady state exercise, thus adjusting the accumulated training load used for the input value in the model.

## Supporting information

S1 Fig Quantification of training load in cycling. An example of monthly cumulative training loads calculated using TRIMP, TSS, and SS (A) or SS_CP_, SS_W′_, and SS_Pmax_ (B). The data were provided by a category 3 female cyclist.
(TIF)

S2 Fig An example of results of fitting the 3D impulse-response model with an athlete’s real-life training and racing data. A) Separate exponentially weighted-moving average fitting performed for CP, W’, and Pmax. The time constants, τ_1_ and τ_2_, for CP were 52 and 10 days, for W′ were 5 and 5 days, and for Pmax were 10 and 4 days, respectively, while k_1_ and k_2_ were 1.6 and 0.6 for CP, 2500 and 2000 for W′, and 51 and 6 for Pmax, respectively. B) A comparison of impulse-response modeling of critical power (CP) using three different training load metrics: total strain score (SS), training stress score (TSS), and training impulse (TRIMP).
(TIF)

S1 File Supplementary material. Outline of the data analysis for n = 1 example cyclist when implementing the 3D impulse-response model.
(DOCX)

S2 File Training dataset. Data used for example calculation in S1 File.
(XLSX)
