# Supplementary material for: The three-dimensional impulse-response model: Modeling the training process in accordance with energy system-specific adaptation
Source: PLoS One. 2026 Feb 6;21(2):e0341721. doi: 10.1371/journal.pone.0341721 (PMC12880663; doi:10.1371/journal.pone.0341721)
Supplement: S1 File — Outline of the data analysis for n = 1 example cyclist when implementing the 3D impulse-response model. (DOCX) [file pone.0341721.s003.docx]

**Supplementary material**

**The three-dimensional impulse-response model: Modeling the training process in accordance with energy system-specific adaptation**

Hilkka Kontro^1*^, Armando Mastracci^2^, Stephen S. Cheung^3^, and Martin J. MacInnis^1^

1 Faculty of Kinesiology, University of Calgary, Alberta, Canada

2 Baron Biosystems Ltd., Toronto, Ontario, Canada

3 Department of Kinesiology, Brock University, Ontario, Canada

**Purpose**

This document outlines the data analysis for n=1 example cyclist for readers interested in implementing the 3D impulse-response model.

**Training data processing**

All data were provided by the athlete (local category 3 female cyclist) as .FIT files. A total of 389 files were processed into .csv files for further analysis.

**Parameter fitting**

Best MMP values were recorded for each month. Highest MMP values for the durations 5, 30, 60, 120, 150, 180, 360, 480, 600, 720, and 1200 seconds were used to fit two 3-parameter models: the original 3P model (3Porig) (Morton, 1996) and the modified 3P model (3Pmod) (Kontro et al., 2024). The parameters (CP, Wʹ, and Pmax) were extracted from the results for each month.

For 3Porig, mean CP = 220.1 W (SD 11.5 W, range 200.4 to 239.6 W); mean Wʹ = 13.8 kJ (SD 5.1 kJ, 6.5 to 20.4); mean Pmax = 846 W (SD 92 W, range 720 to 1072 W). For 3Pmod: mean CP = 225.3 W (SD 11.1 W, range 203.9 to 245.1 W); mean Wʹ = 11.3 kJ (SD 3.6 kJ, range 6.0 to 15.6); mean Pmax = 745 W (SD 56 W, range 641 to 859 W). SE for 3Pmod was lower for all parameters compared to 3Porig (P < 0.001, mean SE 5.6 vs. 7.2 W for CP, 1.5 vs. 0.8 kJ for Wʹ, and 12 vs. 21 W for Pmax); therefore, 3Pmod was selected as the model for training load analysis. Of note, the selection of models for the training load analysis only has a minor impact on SS scores given the close proximity of the parameters obtained by each model.

**Training load analysis**

SS_CP_, SS_Wʹ_, and SS_Pmax_ were calculated for each month using the most current parameters using Equations 8-13. TSS was calculated using the most current CP using Equation 1 (Coggan, 2014). Banister’s TRIMP was calculated using athlete’s reported resting HR (45 bpm) and the highest HR value in the data as maximal HR (190 bpm), and the female-specific constant 1.67 (Morton et al., 1990)

Power data were estimated from HR in files missing power (n=31) using linear equation derived from data points with steady state efforts. Similarly, TRIMP was estimated from power in files missing HR (n=18) using the same HR-power relationships. No files were missing both power and HR. Fig S1 illustrates the monthly cumulative training loads calculated using TRIMP, TSS, and SS (A) or SS_CP_, SS_Wʹ_, and SS_Pmax_ (B).





**Fig S1.**

**3D impulse-response modeling**

The exponentially weighted-moving average approach was used to find the relationship between SS scores and observed parameters to the 3D impulse-response model. The model parameters were estimated iteratively using nonlinear least-squares (Levenberg–Marquardt) optimization. To ensure physiologically realistic solutions, each parameter was confined within predefined lower and upper limits using a logistic re-parameterization (“boxing”) approach. In this method, parameters were optimized in an unconstrained space and continuously mapped to their bounded values through a smooth logistic transform, guaranteeing that all estimates remained within the specified ranges throughout the iterations. The algorithm updated parameters until successive iterations produced negligible changes in the residual sum of squares. The results are shown in Fig S2.

It is important to note that this analysis is for illustration purposes, and the observed data are not collected with standardized mean maximal power tests, and therefore it is unsurprising that the prediction capability of the models is poor in some instances. For example, best 5-s power values for most months were derived from later stages in a virtual or outdoor race and therefore not representative of sprint ability in optimal conditions. A prospective validation study spanning several months and including frequent standardized testing is required to better evaluate the accuracy of this model.

Using TSS or TRIMP instead of SS didn’t not substantially improve the model’s prediction of CP evolution over the training year (Fig S2). However, these metrics lack the potential to also predict the evolution of W’ and Pmax.





**Fig S2.**

**Code availability**

The code used to analyze these example data is available on GitHub: <https://github.com/HKont/3DIR-model-code> .

**References**

Coggan, A. (2014). *What is TSS? By TrainingPeaks*. Retrieved 24/11/2020 from <https://www.trainingpeaks.com/blog/normalized-power-intensity-factor-training-stress/>

Kontro, H., Mastracci, A., Cheung, S., & MacInnis, M. J. (2024). Maximum Power Available: An Important Concept for Prediction of Task Failure and Improved Estimation of Training Loads in Cycling. *Journal of Science and Cycling*, *13*(2), 7-9.

Morton, R. H. (1996). A 3-parameter critical power model. *Ergonomics*, *39*(4), 611-619. <https://doi.org/10.1080/00140139608964484>

Morton, R. H., Fitz-Clarke, J. R., & Banister, E. W. (1990). Modeling human performance in running. *J Appl Physiol (1985)*, *69*(3), 1171-1177. <https://doi.org/10.1152/jappl.1990.69.3.1171>
